# Dentoskeletal features and growth pattern in Beckwith-Wiedemann spectrum: is surgical tongue reduction always necessary?

**DOI:** 10.1007/s00784-023-05043-w

**Published:** 2023-05-10

**Authors:** Patrizia Defabianis, Rossella Ninivaggi, Federica Romano

**Affiliations:** Department of Surgical Sciences, Section of Pediatric Dentistry, C.I.R. Dental School, University of Turin, Via Nizza 230, 10126 Turin, Italy

**Keywords:** Beckwith–Wiedemann syndrome, Craniofacial growth pattern, Glossectomy, Macroglossia, Tongue reduction surgery, Malocclusion

## Abstract

**Objectives:**

The role of tongue reduction surgery (TRS) in preventing excessive mandibular growth and anterior open bite in children with Beckwith–Wiedemann Spectrum (BWSp) is still controversial. This cross-sectional study aimed at comparing craniofacial growth pattern in children affected by BWSp either treated or not treated with early TRS for severe macroglossia. Considering the invasive nature of such surgery, the present study could help in clarifying the need for TRS to reduce or prevent growth disturbances.

**Materials and methods:**

Orthopantomography and lateral skull x-ray images were taken either from surgically treated or non-surgically treated patients, aged 5 to 8 years, to compare dentoskeletal features and craniofacial growth by cephalometric analysis. Molecular testing results were collected from their medical records.

**Results:**

Eighteen BWSp patients were consecutively recruited: 8 underwent TRS at 14.9 ± 2.2 months of age, while 10 did not. Anterior open bite and dental class III were more frequently observed in the surgically treated group, but none showed skeletal class III. No statistically significant differences were observed in growth pattern, but children treated with TRS showed a tendency towards both maxillary and mandibular prognathism with protruding lower lip. Growth pattern seemed to be not related to molecular subtypes.

**Conclusions:**

These preliminary data suggest that early TSR does not improve craniofacial growth pattern and dentoskeletal features in BWSp children.

**Clinical relevance:**

Reductive glossectomy may not be justified for preventing or avoiding oro-facial deformities in BWSp; therefore, early monitoring of maxillofacial development of each affected child has a great clinical significance.

**Supplementary Information:**

The online version contains supplementary material available at 10.1007/s00784-023-05043-w.

## Introduction

Beckwith-Wiedemann Syndrome (BWS) (OMIM#130650) is a rare overgrowth disorder due to (epi)genetic alterations in growth-regulating genes on chromosome 11p15 [1]. Given the complexity of phenotypic expression and (epi)genotypic anomalies, it has been reclassified in 2018 as Beckwith-Wiedemann spectrum (BWSp) [2]. Its main phenotypic manifestations include exompholos, macroglossia, visceromegaly, and cancer predisposition [3, 4].

Macroglossia is a cardinal trait of BWSp, affecting 80–99% of cases [2]. It results from the hyperplasia of skeletal muscle fibers, with variable phenotypic features and degrees of severity [5]. As a consequence, the standardization of its treatment is still challenging. In the neonatal period, tongue reduction surgery (TRS) is essential in case of impairment of vital functions such as airway obstruction and swallowing problems with failure to thrive due to extremely severe macroglossia [6, 7]. Otherwise, glossectomy is usually delayed until after the first year of life if functional deficits (sialorrhea, dysphagia, sleep apnea, and language delay) are still present [8, 9]. Prevention of dentofacial deformities is another accepted indication for early TRS [8, 10]. Many authors advocated that the increased pressure of the tongue on frontal teeth might result in oro-facial growth disturbances [8, 11]. Nonetheless, the role of TRS in preventing dentoskeletal alterations is not fully elucidated yet. Indeed, some studies suggested that TRS prevents mandibular prognathism and open bite [10, 12, 13], while others report similar dentoalveolar alterations and facial growth patterns either in treated or untreated children [14, 15].

Therefore, the aim of this cross-sectional study was to compare the dentoskeletal features and craniofacial growth between untreated and treated BWSp patients with early TRS. This may contribute to clarify the need of such surgical procedure in controlling the mandibular growth and the development of class III skeletal malocclusion.

## Materials and methods

### Study design and patient selection

This study was designed as a prospective, cross-sectional, single-center study. The protocol was approved by the Institutional Ethics Committee (protocol number 1103–2019) and complies with the ethical principles of the Helsinki declaration. All parents/guardians provided written informed consent before children enrolment.

Children with clinical diagnosis of BWSp [2] were consecutively recruited among those referred by pediatric geneticists from Children Hospital to the Section of Pediatric Dentistry of the University of Turin from September 2019 to January 2022. Only patients with severe macroglossia requiring early TRS, regardless of whether they had undergone one or more surgical interventions or not, were enrolled. Furthermore, only children aged between 5 and 8 years at the time of the dental visit were included in the study, while subjects younger than 4 and/or with a history of previous orthodontic treatment were excluded.

### Surgical treatment

The diagnosis of macroglossia was based on subjective clinical criteria, and it referred to a tongue protruding beyond the teeth and the alveolar ridge in resting position [16]. Considering the size of the patients’ tongue and the associated functional deficits, such as difficulties in management of saliva (drooling), swallowing, or speech difficulties, early TRS using the keyhole technique was proposed to all parents. This combined procedure involves an anterior wedge excision and a posterior V-shaped drawing, allowing a uniform tongue reduction, while preserving its neurovascular structure [17]. The same surgeon explained the advantages and disadvantages of the surgical intervention and the risk for intraoperative and postoperative complications. Patients were also informed about the possibility that the lingual size might gradually decrease and the oral cavity might accommodate the tongue with the growth of children [4].

### Data collection

**Fig. 1:**
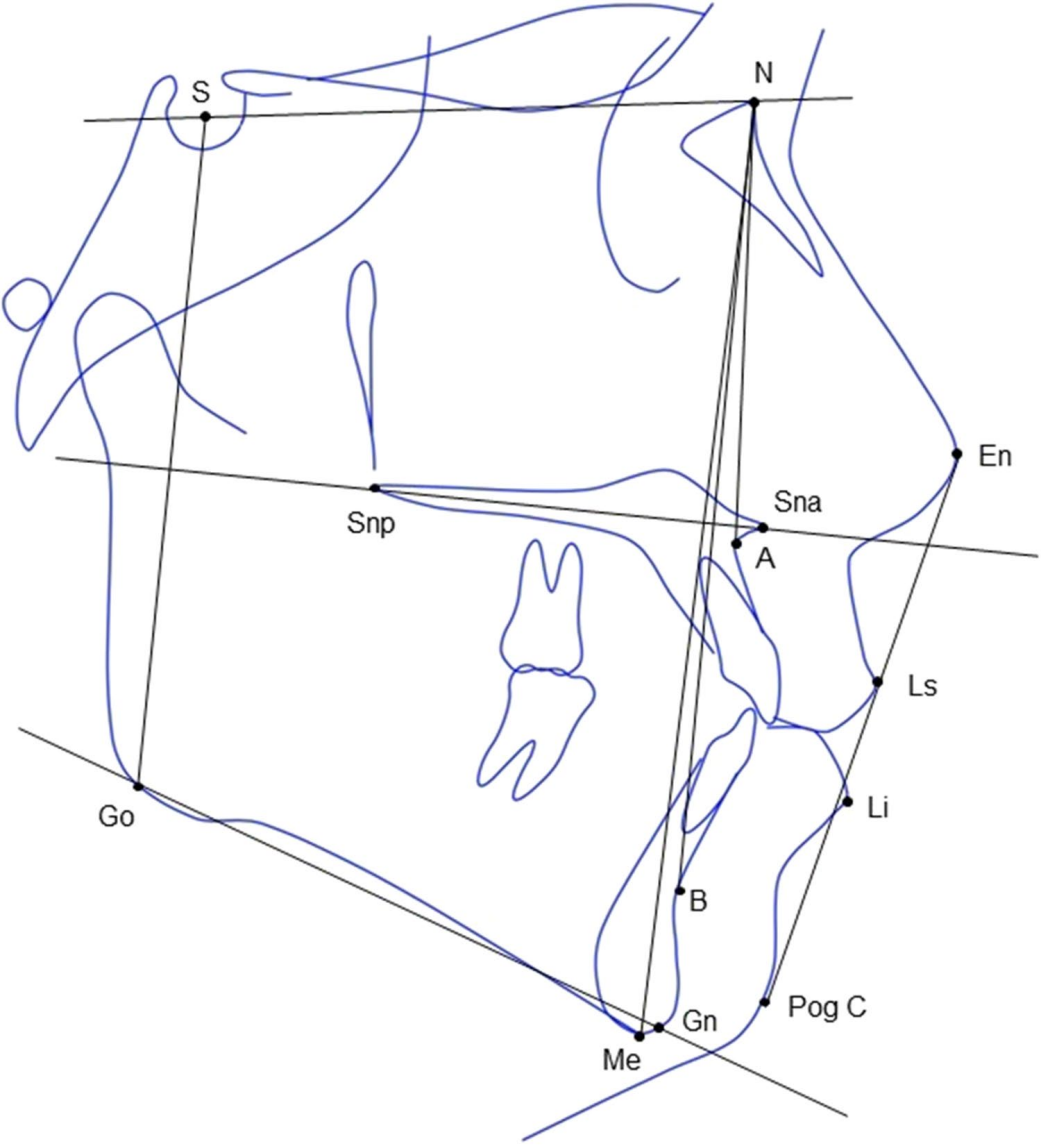
Cephalometric landmarks and lines used in the present study. A total of 13 landmarks were included in the analysis: N, nasion; A, point A (most concave point in the anterior maxilla); B, point B (most concave point of mandibular symphysis); S, sella (mid-point of sella turcica); Sna, anterior nasal spine; Snp, posterior nasal spine; Me, menton (most inferior point of mandibular symphysis); Go, gonion (midpoint of the mandibular angle); Gn, Gnathion (most anterior-inferior point of the bony chin); En, pronasale (most prominent point of the nose); Li, labialis inferior (most anterior point on the lower lip); Ls, = labialis superior (most anterior point on the upper lip); PogC, soft tissue pogonion (most anterior point on the soft tissue chin). The lines connecting the landmarks were as follows: N and A point (NA line), N and B point (NB line), S and N (SN line), Gn and Go (Mandibular plane), S and Go (S-Go line), N and Me (N-Me line), soft tissue PogC and soft tissue En (E-line), Snp and Sna (Palatal Plane)

Data collected included demographics, results of genetic tests, clinical symptoms, type of surgery performed, age at which surgery was performed, need for secondary reduction surgery, referral of speech, and/or taste difficulties after TRS. Orthopantomography and lateral skull x-ray images were taken from all patients to evaluate dentoskeletal features and growth tendency. Traditional cephalometric landmarks and linear and angular dimensions (Fig. 1) were traced according to the Björk-Jarabak method [18, 19] by a single specialist in pediatric dentistry; all were repeated twice, with a 4-week interval in between, to minimize errors as described in literature [11]. The measurements recorded were as follows:

- Antero-posterior skeletal pattern: class I = ANB < 0°; class II = ANB between 0° and 4°; class III = ANB > 4°; vertical skeletal pattern: normal = SpP^GoGn between 15°and 25°; hypodivergent = SpP^GoGn < 15°; hyperdivergent = SpP^GoGn > 25°. These parameters were recorded to evaluate the mandibular position in relation to the maxillary one.
- Maxillary position: normal = SNA between 80° and 84°; maxillary retrognathism = SNA < 80°; maxillary prognathism = SNA > 84°; mandibular position: normal = SNB between 78° and 82°; mandibular retrognathism = SNB < 78°; mandibular prognathism = SNB > 82°. These data gave information about the protruded/retruded position of both maxilla and mandible.
- Vertical growth pattern based on the ratio expressed in percentage between the posterior (S-Go) and the anterior facial height (N-Me) according to Jarabak [19]: clockwise growth = S-Go/N-Me < 62%; straight-down growth = S-Go/N-Me between 62 and 65%; counter clockwise growth = S-Go/N-Me > 65%. These data gave information about the growth tendency: in a clockwise growth, the mandible grows anteriorly and upward; in a counterclockwise growth, the mandible grows backward and downward.
- Upper lip profile: normal = Ls-E between − 4 and 0 mm; retrusion = Ls-E <  − 4 mm; protrusion Ls-E > 0 mm; lower lip profile: normal = Li-E between − 4 and 0 mm; retrusion = Li-E <  − 4 mm; protrusion Li-E > 0 mm. These parameters gave information on the influence of dentoskeletal features on esthetics.

Children were also scored with a composite prognostic index based on vertical growth pattern in relation to the intermaxillary divergence [20].

### Statistical analysis

Quantitative variables were expressed as mean ± standard deviation (SD) or median and interquartile range (IQR), while categorical variables were presented as frequencies. The statistical significance of the differences between children treated and not treated with TRS was evaluated using independent *t* test (for normally distributed variables) and Mann–Whitney *U* test (for non-normally distributed variables). Chi-square and Fisher’s exact test were applied for group comparisons of qualitative data. Significance level was set at 5%, and the Statistical Package for the Social Sciences (IBM SPSS version 25.0, USA) was used for data analysis.

## Results

A total of 34 Caucasian BWSp patients were screened and 18/34 met the inclusion criteria. All showed a severe macroglossia at birth, but none of them experienced either feeding or breathing life-threatening complications requiring TRS during the first months of life. Eating with the tongue protruding beyond the lips and anterior spillage of food or fluid from oral cavity and wetting clothing were the main concerns reported by parents before surgery. Among the enrolled children, 8/18 children (3 males and 5 females, 6.4 ± 1.3 years) underwent TRS (treated group) at 14.9 ± 2.2 months of age (range 12–19 months). The same maxillofacial surgeon performed surgery using a keyhole technique. None of the children required repeated surgery to achieve an appropriate tongue reduction. The remaining 10 patients (5 males and 5 females, 6.2 ± 1.2 years) did not undergo TRS because the parents declined surgery (untreated group).

No difference was found between the two groups in terms of gender distribution, age and frequency of molecular genotypes (Supplementary Table 1). However, gain of methylation at imprinting centers 1 (IC1-GoM) was observed only in the treated group. All children showed an intraoral position of the tongue at the time of the cephalometric analysis, regardless of whether had undergone early TRS. Anterior open bite and dental class III were more frequently observed in the surgically treated group (*p* = 0.06); none of the 18 patients included in the study showed a skeletal class III, while skeletal classes I and II were evenly distributed between the two groups. No statistically significant difference in growth tendency and prognosis was observed, but 2/18 patients (one in the treated and one in the untreated group) showed a counter clockwise growth pattern. Regarding cephalometric data, children treated with TRS showed significantly higher values of SNA, SNB, and Li-E compared with the untreated controls (*p* < 0.05) (Table 1).

**Table 1:** Maxillofacial morphology, growth pattern, and cephalometric measurements according to tongue reduction surgery

	Group			
Variables	Treated (*N* = 8)	Untreated (*N* = 10)	Total (*N* = 18)	*P-*value
Dental class, *n* (%)				0.065
I	5 (38.5)	8 (61.5)	13 (72.2)	
II	0 (0.0)	2 (100.0)	2 (11.1)	
III	3 (100.0)	0 (0.0)	3 (16.7)	
Skeletal class (ANB), *n* (%)				1.000
I	4 (40.0)	6 (60.0)	10 (55.6)	
II	4 (50.0)	4 (50.0)	8 (44.4)	
III	0 (0.0)	0 (0.0)	0 (0.0)	
Divergence (SpP^GoGn), *n* (%)				0.867
Normal	1 (50.0)	1 (50.0)	2 (11.1)	
Hyperdivergence	7 (56.3)	9 (43.8)	16 (88.9)	
Hypodivergence	0 (0.0)	0 (0.0)	0 (0.0)	
Growth pattern (S-Go/N-Me), *n* (%)				0.789
Clockwise	3 (60.0)	2 (40.0)	5 (27.8)	
Straight-down	4 (36.4)	7 (63.6)	14 (61.1)	
Counter clockwise	1 (50.0)	1 (50.0)	2 (11.1)	
Open bite, *n* (%)	7 (55.6)	4 (34.6)	11 (61.1)	0.066
Prognostic score, *n* (%)				0.427
Positive	0 (0.0)	1 (100)	1 (5.6)	
Negative	2 (40.0)	3 (60.0)	5 (27.8)	
Neutral	8 (66.7)	4 (33.3)	12 (66.7)	
SNA (°), mean ± SD	86.9 ± 5.1	81.3 ± 2.4		0.020
SNB (°), mean ± SD	81.5 ± 5.6	76.9 ± 2.1		0.028
ANB (°), median (IQR)	4.5 ± 3.3	4.0 (1.5)		0.696
SpP^GoGn (°), mean ± SD	31.1 ± 5.6	27.8 ± 2.5		0.118
S-Go (mm), mean ± SD	66.1 ± 10.8	66.4 ± 7.3		0.939
N-Me (mm), median (IQR)	106.5 (13.7)	107.5 (5.6)		0.997
S-Go/N-Me (%), mean ± SD	61.6 ± 3.2	62.9 ± 3.6		0.409
Ls-E line (mm), mean ± SD	1.7 ± 1.5	1.0 ± 2.1		0.425
Li-E line (mm), mean ± SD	3.3 ± 2.3	1.3 ± 1.7		0.049

For cephalometric measurements, refer to the legend of Fig. 1

*SD* standard deviation, *IQR* interquartile range

An exploratory analysis was done to assess the distribution of growth tendency and molecular subtypes in relation to tongue surgery. A trend in the data was not apparent, although a statistical analysis was not performed due to the small sample size.

## Discussion

The present study showed that early TRS has no impact on craniofacial growth and dentoskelatal morphology. Early surgery performed in patients younger than 2 years of age using the keyhole technique resulted in growth patterns similar to those of untreated controls, with the exception of more frequent maxillary and mandibular protrusion. This surgical procedure decreases the tongue size in all dimensions, meanwhile preserving its neurovascular structure, mobility, and appearance [21, 22]. It improves speech and articulation, but it negatively impacts on taste perception due to the loss of the tongue tip, even if no marked sensory deficit has been described [23–25]. The average age at surgery was 14.5 ± 2.1 months, which is consistent with the range of most studies [22, 26].

Although macroglossia has often been related to skeletal class III malocclusion and downward mandibular rotation [11], none of the enrolled children showed skeletal class III growth pattern and only two of them exhibited counter clockwise growth tendency. Consistent with a previous study, no association between maxillofacial phenotype and (epi)genotype emerged [20].

According to some authors, TRS in BWSp children is aimed at controlling facial growth as early glossectomy should minimize the impact of macroglossia on it [8, 10, 27], reducing the time and complexity of later orthodontic treatment and/or orthognathic surgery [12, 13, 26]. Anyway, data reported in literature are difficult to compare due to the lack of guideline and validated scoring criteria for the severity of macroglossia and standardized timing for surgery. Furthermore, most of the studies reporting favorable craniofacial outcomes after TRS are case reports or case series on patients of different race/ethnicity [10, 12, 13]. Currently, there are only few studies on Caucasian BSWp children that compared surgically treated patients and untreated controls and all reported similar dentoalveolar alterations and mandibular growth regardless of TRS [8, 15]. Consistently, Kawafuji et al. speculated that the enlargement of the mandibular body might be due not to the macroglossia, but to the IGF2 effect, suggesting so that it might be a facial trait of the syndrome [11].

This study supports such findings: although TRS is essential in treating BWSp patients in case of life-threatening conditions, such as upper air obstruction and poor nutritional status, reductive glossectomy aimed at preventing mandibular growth seems not to be justified. TRS is an invasive procedure and either patients/parents/caregivers have expressed concerns about outcomes, especially with respect to taste, tongue function, and appearance [28].

These data should be interpreted within the limitations of the current study that enrolled only Caucasian children who underwent TRS younger than 2 years of age using the keyhole procedure. However, it should be taken into consideration that all children were referred from a single center in North Italy and were treated by the same pediatric surgeon. It is also possible that cases diagnosed as severe macroglossia had varying tongue dimensions and lengths of protrusion due to the lack of an objective method for a consistent assessment of tongue appearance and function. Anyway, the two groups of children were matched by the same clinical indications for TRS.

Furthermore, the number of children was small, even if consistent with the sample size of other studies due to the rarity of BWSp, and this could have resulted in a reduction of the statistical power of our study. That is why we need more investigation on larger samples of affected individuals, together with a precise record of the extent and size of the macroglossia and glossectomy. This is not only to standardize criteria for patients’ selection, but also for timing the procedures; for all these reasons, follow-up studies to optimize long-term results and to improve the overall quality of life of children with BWSp [29] are required.

## Supplementary Information

Below is the link to the electronic supplementary material.

Supplementary file 1 (DOCX 30 KB)

## Data Availability

The data underlying this article cannot be shared publicly due to the vulnerable study population. The data will be shared on reasonable request to the corresponding author.

## References

[1] Fontana L, Tabano S, Maitz S, Colapietro P, Garzia E, Gerli AG, Sirchia SM, Miozzo M (2021). Clinical and molecular diagnosis of Beckwith-Wiedemann syndrome with single- or multi-locus imprinting disturbance. Int J Mol Sci.

[2] Brioude F, Kalish JM, Mussa A (2018). Expert consensus document: clinical and molecular diagnosis, screening and management of Beckwith-Wiedemann syndrome: an international consensus statement. Nat Rev Endocrinol.

[3] Mussa A, Di Candia S, Russo S, Catania S, De Pellegrin M, Di Luzio L, Ferrari M, Tortora C, Meazzini MC, Brusati R, Milani D, Zampino G, Montirosso R, Riccio A, Selicorni A, Cocchi G, Ferrero GB (2016). Recommendations of the Scientific Committee of the Italian Beckwith-Wiedemann Syndrome Association on the diagnosis, management and follow-up of the syndrome. Eur J Med Genet.

[4] Friede H, Figueroa A (1985). The Beckwith-Wiedemann syndrome: a longitudinal study of the macroglossia and dentofacial complex. J Craniofac Genet Dev Biol Suppl.

[5] Oyama Y, Nishida H, Kobayashi O, Kawano K, Ihara K, Daa T (2020). Macroglossia in Beckwith-Wiedemann syndrome is attributed to skeletal muscle hyperplasia. Case Rep Dent.

[6] Naujokat H, Möller B, Terheyden H, Birkenfeld F, Caliebe D, Krause MF, Fischer-Brandies H, Wiltfang J (2019). Tongue reduction in Beckwith-Wiedemann syndrome: outcome and treatment algorithm. Int J Oral Maxillofac Surg.

[7] Rimell FL, Shapiro AM, Shoemaker DL, Kenna MA (1995). Head and neck manifestations of Beckwith-Wiedemann syndrome. Otolaryngol Head Neck Surg.

[8] Simmonds JC, Patel AK, Mader NS, Scott AR (2018). National trends in tongue reduction surgery for macroglossia in children. J Craniomaxillofac Surg.

[9] Follmar A, Dentino K, Abramowicz S, Padwa BL (2014). Prevalence of sleep-disordered breathing in patients with Beckwith-Wiedemann syndrome. J Craniofac Surg.

[10] Matsuda H, Tamura H, Tonoki M (2017). Efficacy and optimal timing of tongue reduction surgery in three patients with Beckwith-Wiedemann syndrome. J Oral Maxillofac Surg Med Pathol.

[11] Kawafuji A, Suda N, Ichikawa N, Kakara S, Suzuki T, Baba Y, Ogawa T, Tsuji M, Moriyama K (2011). Systemic and maxillofacial characteristics of patients with Beckwith-Wiedemann syndrome not treated with glossectomy. Am J Orthod Dentofacial Orthop.

[12] Alonso-Rodriguez E, Gómez E, Martín M, Muñoz JM, Hernández-Godoy J, Burgueño M (2018). Beckwith-Wiedemann syndrome: open bite evolution after tongue reduction. Med Oral Pat Oral Cir Bucal.

[13] Harada T, Yamanishi T, Kurimoto T, Nishio J (2019). Improved quality of life for children with Beckwith-Wiedemann syndrome following tongue reduction surgery. J Craniofac Surg.

[14] Naujokat H, Möller B, Terheyden H, Birkenfeld F, Caliebe D, Krause MF, Fischer- Brandies H, Wiltfang J (2018). Tongue reduction in Beckwith-Wiedemann syndrome: outcome and treatment algorithm. Int J Oral Maxillofac Surg.

[15] Meazzini MC, Besana M, Tortora C, Cohen N, Rezzonico A, Ferrari M, Autelitano L (2020). Long-term longitudinal evaluation of mandibular growth in patients with Beckwith-Wiedemann syndrome treated and not treated with glossectomy. J Cranio-Maxillofacial Surg.

[16] Vogel JE, Mulliken JB, Kaban LB (1986). Macroglossia: a review of the condition and a new classification. Plast Reconstr Surg.

[17] Morgan WE, Friedman EM, Duncan NO, Sulek M (1996) Surgical management of macroglossia in children. Arch Otolaryngol – Head Neck Surg 122:326–329. 10.1001/archotol.1996.01890150096017 10.1001/archotol.1996.01890150096017 8607962

[18] Björk A (1969). Prediction of mandibular growth rotation. Am J Orthod.

[19] Jarabak JR, Fizzel JA (1972). Technique and treatment with light wire edgewise appliances.

[20] Defabianis P, Mussa A, Ninivaggi R, Carli D, Romano F (2022). Maxillo-facial morphology in patients with Beckwith-Wiedemann syndrome: a preliminary study on (epi)genotype phenotype association in Caucasians. Int J Environ Res Public Health.

[21] Kacker A, Honrado C, Martin D, Ward R (2000). Tongue reduction in Beckwith-Weidemann syndrome. Int J Pediatr Otorhinolaryngol.

[22] Cohen JL, Cielo CM, Kupa J, Duffy KA, Hathaway ER, Kalish JM, Taylor JA (2020). The utility of early tongue reduction surgery for macroglossia in Beckwith-Wiedemann syndrome. Plast Reconstr Surg.

[23] Maas SM, Kadouch DJ, Masselink AC, Van Der Horst CM (2016). Taste and speech following surgical tongue reduction in children with Beckwith-Wiedemann syndrome. J Craniomaxillofac Surg.

[24] Balaji SM (2013). Reduction glossectomy for large tongues. Ann Maxillofac Surg.

[25] Kaufman Y, Cole P, McKnight A, Hatef DA, Hollier L, Edmonds JA (2008). Modified keyhole technique for correction of macroglossia. Plast Reconstr Surg.

[26] Kadouch DJ, Maas SM, Dubois L, van der Horst CM (2012). Surgical treatment of macroglossia in patients with Beckwith-Wiedemann syndrome: a 20-year experience and review of the literature. Int J Oral Maxillofac Surg.

[27] Alonso-Rodriguez E, Gómez E, Martín M, Muñoz JM, Hernández-Godoy J, Burgueño M (2018). Beckwith-Wiedemann syndrome: open bite evolution after tongue reduction. Med Oral Patol Oral Cir Bucal.

[28] Tomlinson JK, Morse SA, Bernard SPL, Greensmith AL, Meara JG (2007). Long-term outcomes of surgical tongue reduction in Beckwith-Wiedemann syndrome. Plast Reconstr Surg.

[29] Defabianis P, Ninivaggi R, Romano F (2022). Oral health-related quality of life among children and adolescents with Beckwith-Wiedemann syndrome in Northern Italy. J Clin Med.

