# Supplementary material for: Dentoskeletal features and growth pattern in Beckwith-Wiedemann spectrum: is surgical tongue reduction always necessary?
Source: Clin Oral Investig. 2023 May 10;27(8):4271–7. doi: 10.1007/s00784-023-05043-w (PMC10415414; doi:10.1007/s00784-023-05043-w)
Supplement: Supplementary file 1 — (DOCX 30 KB) [file 784_2023_5043_MOESM1_ESM.docx]

**Dentoskeletal features and growth pattern in Beckwith-Wiedemann spectrum: is surgical tongue reduction always necessary?**

Patrizia Defabianis,^1^ Rossella Ninivaggi,^1^ Federica Romano,^1^

^1^Department of Surgical Sciences, C.I.R. Dental School, University of Turin, Turin, 10126, Italy

Corresponding Author: Patrizia Defabianis E-mail: patrizia.defabianis@unito.it

**Table 1.** Demographic, genetic and oral features of patients with BWS according to tongue reduction surgery.

|  | **Group** | | |  |
| --- | --- | --- | --- | --- |
| **Variables** | **Treated**  ***(*n = 8)** | **Untreated**  **(n = 10)** | **Total**  **(n = 18)** | **P*-*value** |
| **Age (years), mean ± SD** | 6.4 ± 1.3 | 6.2 ± 1.2 | 6.3 ± 1.2 | 0.728 |
| **Sex, female/male** | 3/5 | 5/5 | 8/10 | 0.664 |
| **Molecolar genotype, n (%)** |  |  |  | 0.589 |
| UPD(11)pat | 2 (40.0) | 3 (60.0) | 5 (27.8) |  |
| IC2-LoM | 2 (33.3) | 4 (66.7) | 6 (33.3) |  |
| IC1-GoM | 2 (100.0) | 0 (0.0) | 2 (11.1) |  |
| Test negative | 2 (40.0) | 3 (60.0) | 5 (27.8) |  |
| **Agenesis, n (%)** | 1 (100.0) | 0 (0.0) | 1 (5.6) | - |
| **dmft, median (IQR)** | 0.5 (5.75) | 2.0 (6.25) | 1.5 (6.0) | 0.633 |
| **Professional oral hygiene frequency, n (%)** |  |  |  | 1.000 |
| At least once/year | 4 (40.0) | 6 (60.0) | 10 (55.6) |  |
| Occasionally | 4 (50.0) | 4 (50.0) | 8 (44.4) |  |
| **Phonation difficulties, *n* (%)** | 0 (0.0) | 1 (100.0) | 1 (5.6) | - |
| **Atypical deglutition, *n* (%)** | 3 (42.9) | 4 (57.1) | 7 (38.9) | 0.914 |

Abbreviations: dmft, decayed missing filled primary teeth index; IC2-LoM, loss of methylation at imprinting centre 2; IC1-GoM, gain of methylation at imprinting centre 2; UPD(11)pat, mosaic paternal uniparental isodisomy of chromosome 11.
